# Ankle-brachial index and incident diabetes mellitus: the atherosclerosis risk in communities (ARIC) study

**DOI:** 10.1186/s12933-016-0476-4

**Published:** 2016-12-07

**Authors:** Simin Hua, Laura R. Loehr, Hirofumi Tanaka, Gerardo Heiss, Josef Coresh, Elizabeth Selvin, Kunihiro Matsushita

**Affiliations:** 1 Department of Epidemiology, Johns Hopkins Bloomberg School of Public Health, Welch Center for Prevention, Epidemiology and Clinical Research, 2024 E. Monument Street Suite 2-600, Baltimore, MD 21287 USA; 2 Department of Epidemiology, The University of North Carolina at Chapel Hill Gillings School of Global Public Health, 137 East Franklin Street, Suite 306, Chapel Hill, NC 27514 USA; 3 Department of Kinesiology & Health Education, The University of Texas at Austin, 2109 San Jacinto Blvd, Austin, TX 78712-1415 USA

**Keywords:** Ankle-brachial index, Peripheral artery disease, Diabetes mellitus, Community-based study, Prospective cohort study

## Abstract

**Background:**

Individuals with peripheral artery disease (PAD) often have reduced physical activity, which may increase the future risk of diabetes mellitus. Although diabetes is a risk factor for PAD, whether low ankle-brachial index (ABI) predates diabetes has not been studied.

**Methods:**

We examined the association of ABI with incident diabetes using Cox proportional hazards models in the ARIC Study. ABI was measured in 12,247 black and white participants without prevalent diabetes at baseline (1987–1989). Incident diabetes cases were identified by blood glucose levels at three subsequent visits (1990–92, 1993–95, and 1996–98) or self-reported physician diagnosis or medication use at those visits or during annual phone interview afterward through 2011.

**Results:**

A total of 3305 participants developed diabetes during a median of 21 years of follow-up. Participants with low (≤0.90) and borderline low (0.91–1.00) ABI had 30–40% higher risk of future diabetes as compared to those with ABI of 1.10–1.20 in the demographically adjusted model. The associations were attenuated after further adjustment for other potential confounders but remained significant for ABI 0.91–1.00 (HR = 1.17, 95% CI 1.04–1.31) and marginally significant for ABI ≤ 0.90 (HR = 1.19, 0.99–1.43). Although the association was largely consistent across subgroups, a stronger association was seen in participants without hypertension, those with normal fasting glucose, and those with a history of stroke compared to their counterparts.

**Conclusions:**

Low ABI was modestly but independently associated with increased risk of incident diabetes in the general population. Clinical attention should be paid to the glucose trajectory among people with low ABI but without diabetes.

**Electronic supplementary material:**

The online version of this article (doi:10.1186/s12933-016-0476-4) contains supplementary material, which is available to authorized users.

## Background

Lower extremity peripheral arterial disease (PAD), typically defined by an ankle-brachial index (ABI) < 0.9 [1], affects 8–10 million people in the United States [2]. PAD increases the risk of cardiovascular disease and reduces quality of life due to ischemic leg pain and intermittent claudication [1, 3, 4].

Regardless of leg symptoms, patients with PAD experience functional decline and impairment [5–7], which are shown to result in reduced level of physical activity [8, 9]. For example, a study observed a 20% decline in accelerometer-measured physical activity level in participants with PAD comparing to those without [8]. Since physically inactivity is an important risk factor of diabetes mellitus [10], it is possible that low ABI is associated with the development of diabetes. Furthermore, ABI, an indicator of severity of atherosclerosis in the legs, is found to be associated with microvascular dysfunction in skeletal muscle which is the largest tissue in the body that is insulin-sensitive and central to glucose utilization and metabolic health [11, 12].

However, to the best of our knowledge, the association of ABI with future risk of diabetes has not yet been studied although the opposite direction of association (i.e., diabetes as a risk factor of PAD) is well-known [2, 13, 14]. Therefore, we aimed to investigate whether ABI is independently associated with incident diabetes in a community-based cohort, the Atherosclerosis Risk in Communities (ARIC) Study.

## Methods

### Study population

The ARIC Study is a community-based prospective cohort study of 15,792 individuals aged 45–64 years at baseline. Participants were recruited at baseline examination (visit 1) during 1987–1989 from four US communities: Forsyth County, North Carolina; Jackson, Mississippi; suburbs of Minneapolis, Minnesota; and Washington County, Maryland [15]. The participants were invited for three short-term follow-up examinations at three-year intervals (visits 2 [1990–1992], 3 [1993–1995], and 4 [1996–1998]). They also received annual telephone interview regarding their lifestyle and clinical conditions. The study was approved by the institutional review boards at all centers, and informed consent was obtained from all participants.

Of 15,792 participants, we excluded 2309 participants with prevalent diabetes (defined as self-reported physician diagnosis or treatment of diabetes, fasting blood glucose ≥126 mg/dl or random blood glucose ≥200 mg/dl at baseline) and 17 participants with no information about diabetes status. We further excluded 40 non-white and non-black participants as well as those with missing information on ABI (n = 476) and any covariates at baseline (n = 596), leaving 12,247 participants in our analysis. Of the study population, 76% participants attended all follow-up visits 2 through 4 while 88% attended at least two follow-up visits and 97% attended at least once. Approximately 92% participants responded to annual telephone interview after visit 4 examination.

### Exposure assessment

ABI was defined as a ratio of systolic blood pressure of ankle to that of arm. The ankle and brachial blood pressures were measured by Dinamap Model 1846 SX during ultrasound assessment, an oscillometric device that obtains repeated blood pressure measurement automatically [3, 16]. Before examination, participants were asked to refrain from smoking, vigorous exercise, and drinking coffee, tea, and soft drinks containing caffeine during the night before and the day of examination [16]. Ankle systolic blood pressure was measured four times in a randomly selected leg and the last non-missing value was used as numerator of ABI. Brachial systolic blood pressure was measured twice in the right arm and the first non-missing value was used as denominator of ABI [17]. According to a previous study, the reliability of the ABI based on single ankle and arm systolic blood pressure was 0.61 (95% CI 0.50, 0.70) [18].

### Outcome assessment

The ascertainment of incident diabetes mellitus was based on two elements, self-reported physician diagnosis or treatment of diabetes during visits or phone interview through April 18, 2011 (interview-based definition) and fasting blood glucose ≥126 mg/dl (7.0 mmol/l), random blood glucose ≥200 mg/dl (11.1 mmol/l), or self-reported physician diagnosis or treatment of diabetes during visits 2 through 4 (visit-based definition), as previously done [19]. Participants who did not develop diabetes during follow-up were censored due to death, loss to follow-up, or end of follow-up. To maximize the statistical power, as the primary outcome, we combined these two definitions but also analyzed them separately as a secondary analysis.

### Covariates of interest

Age, gender, race, parental history of diabetes, medical history of coronary heart disease (CHD) and stroke/transient ischemic attack (TIA), smoking and alcohol drinking habits and exertional leg pain were self-reported at baseline. Medication use was assessed by self-report and examination of medication containers brought to the visit. Height, weight, and sitting blood pressure were measured according to standardized protocols. Hypertension was defined as a systolic blood pressure ≥140 mmHg or a diastolic blood pressure ≥90 mmHg, or use of antihypertensive drugs. Total cholesterol level, high-density lipoprotein cholesterol level, and triglyceride level were measured using enzymatic determination methods. Glucose was measured using the hexokinase-glucose-6-phosphate dehydrogenase method, as detailed previously [19]. White blood cell count was measured by automated hematology analyzer [20]. Fibrinogen was measured using assay according to standard procedures [21]. Physical activity was assessed with the Baecke physical activity questionnaire, which recorded the duration, intensity and frequency of physical activity at work, in leisure time and during sports and produced an index score to represent level of physical activity [22].

### Statistical analyses

We categorized ABI into seven groups, ABI ≤ 0.90, 0.90 < ABI ≤ 1.00 (denoted as 0.91–1.00), 1.01–1.10, 1.11–1.20, 1.21–1.30, 1.31–1.40 and >1.40, consistent with prior literature [3]. Baseline characteristics were compared across these groups, according to Chi square test and ANOVA, as appropriate.

To visualize potentially non-linear associations, we estimated incidence rates of diabetes according to ABI with its linear spline terms (knots at 0.9, 1.0, 1.1, 1.2, 1.3 and 1.4) using Poisson regression models. We subsequently quantified the adjusted risk of incident diabetes according to the seven ABI categories using Cox proportional hazards models. ABI 1.11–1.20 was used as the reference group since this group was used as the reference in an international meta-analysis and had the largest number of participants in our study [23]. To evaluate the impact of potential confounding, we constructed three models. Model 1 was adjusted for age, sex and race. Model 2 included all variables in Model 1 plus factors associated with atherosclerosis and diabetes, namely body mass index, total cholesterol, high-density lipoprotein cholesterol, and triglyceride, drinking and smoking status (current, former, and never), systolic blood pressure, hypertension medication use, history of CHD, stroke or TIA, statin use, parental history of diabetes, white blood cell count, and physical activity index. Models 3 and 4 included all variables in Model 2 plus baseline fasting glucose or homeostatic model assessment of insulin resistance (HOMA-IR), respectively. There was no major deviation from the proportional hazards assumption for ABI categories based on visual evaluation from log–log plot as well as from test on Schoenfeld residuals.

To evaluate whether the association is consistent across demographic and clinical subgroups, we tested for interaction and conducted subgroup analyses by age (≤vs. >55 years), gender, race, smoking status (current vs. former/never), history of cardiovascular disease, history of stroke or TIA, hypertension status, baseline fasting glucose level [normal <100 (<5.6 mmol/l) vs. impaired 100–125 mg/dl (5.6–6.9 mmol/l)] and exertional leg pain status. Interaction was tested by incorporating a product term of ABI categories and subgroups in Cox models.

As a sensitivity analysis, we repeated the analysis in visit-based definition and interview-based definition of diabetes separately. We also explored the model which replaced white blood cell count with fibrinogen (an alternative inflammatory marker) in Model 2. Finally, we treated physical activity, a potential mediator of ABI-diabetes association, as a time-varying covariate using self-report data assessed at visit 3 in addition to Model 2 covariates.

All analyses were performed with Stata version 12.0. All p values were two-sided, and p < 0.05 was considered statistically significant.

## Results

The mean ABI of study population was 1.13 (SD 0.14). There were 455 individuals (3.7%) with ABI ≤ 0.90 and 1529 participants (12.5%) with borderline low ABI of 0.91–1.00. There was no significant correlation between ABI and baseline fasting glucose (r = 0.002, p value = 0.81). Baseline characteristics of study participants by ABI categories are shown in Table 1. As compared to participants with ABI 1.11–1.20 (reference group), those with lower ABI were more likely to be older, female, and blacks. They also had worse cardiovascular risk profiles relative to the reference group, including higher prevalence of current smokers, hypertension, and cardiovascular diseases (CHD and stroke/TIA), higher levels of body mass index, total cholesterol, triglyceride, white blood cell count, and fibrinogen, and lower level of physical activity. Participants with ABI > 1.40, indicative of arterial calcification [24], also had worse cardiovascular risk profiles as compared to those with ABI 1.11–1.20. The fasting glucose levels are similar across ABI categories. However, participants with ABI ≤ 0.90 and ABI > 1.40 had higher levels of fasting insulin and HOMA-IR than the rest groups.

**Table 1:** Baseline characteristics of participants without prevalent diabetes by ABI categories

Characteristics	ABI ≤ 0.90	0.91–1.00	1.01–1.10	1.11–1.20	1.21–1.30	1.31–1.40	ABI > 1.40
N (%)	455 (3.7)	1529 (12.5)	2894 (23.6)	3633 (29.7)	2509 (20.5)	924 (7.5)	303 (2.5)
Age, mean (SD), years^a^	54.9 (5.9)	53.7 (5.8)	53.4 (5.6)	53.9 (5.8)	53.9 (5.6)	54.6 (5.7)	55.1 (5.7)
Female, no. (%)^a^	321 (70.6)	1116 (73.0)	1867 (64.5)	1903 (52.4)	1104 (44.0)	373 (40.4)	125 (41.3)
Black, no. (%)^a^	121 (26.6)	357 (23.4)	700 (24.2)	867 (23.9)	542 (21.6)	177 (19.2)	47 (15.5)
BMI, mean (SD), kg/m^2a^	27.7 (6.0)	27.7 (5.8)	27.1 (5.2)	27.0 (4.7)	27.0 (4.6)	27.4 (4.8)	28.3 (5.5)
Height, mean (SD), cm^a^	165.2 (8.9)	165.5 (8.5)	167.0 (9.0)	169.2 (9.3)	170.5 (9.3)	171.4 (9.5)	170.3 (9.5)
Current drinker, no. (%)^a^	244 (53.6)	874 (57.2)	1698 (58.7)	2189 (60.3)	1525 (60.8)	550 (59.5)	163 (53.8)
Current smoker, no. (%)^a^	167 (36.7)	440 (28.8)	798 (27.6)	855 (23.5)	602 (24.0)	195 (21.1)	63 (20.8)
Arm SBP, mean (SD), mmHg^a^	123.0 (19.7)	121.0 (19.2)	120.3 (18.8)	119.7 (17.5)	118.2 (16.6)	118.0 (15.8)	117.6 (15.3)
Heart rate. mean (SD), /min^a^	68.5 (11.4)	67.7 (10.5)	66.9 (9.7)	65.8 (9.6)	64.5 (9.6)	64.3 (9.4)	64.2 (9.8)
Hypertension medication, no. (%)^a^	169 (37.1)	449 (29.4)	810 (28.0)	932 (25.7)	587 (23.4)	221 (23.9)	95 (31.4)
Prevalent CHD, no. (%)^a^	30 (6.6)	68 (4.5)	103 (3.6)	137 (3.8)	103 (4.1)	38 (4.1)	20 (6.6)
History of stroke or TIA, no. (%)^a^	33 (7.3)	68 (4.5)	123 (4.3)	151 (4.2)	99 (4.0)	26 (2.8)	15 (5.0)
Total cholesterol, mean (SD), mmol/l^a^	5.8 (1.1)	5.6 (1.0)	5.6 (1.1)	5.5 (1.1)	5.5 (1.0)	5.5 (1.0)	5.5 (1.1)
HDL, mean (SD), mmol/l^a^	1.4 (0.5)	1.4 (0.5)	1.4 (0.5)	1.4 (0.4)	1.3 (0.4)	1.3 (0.4)	1.3 (0.4)
Triglyceride, median (IQR), mmol/l^a^	1.3 (0.9–1.8)	1.2 (0.9–1.6)	1.2 (0.8–1.6)	1.2 (0.9–1.7)	1.2 (0.9–1.7)	1.2 (0.9–1.7)	1.3 (0.9–1.8)
Statin use, no. (%)	5 (1.1)	13 (0.9)	16 (0.6)	12 (0.3)	9 (0.4)	5 (0.5)	2 (0.7)
Fasting glucose, mean (SD), mmol/l	5.5 (0.5)	5.5 (0.5)	5.5 (0.5)	5.5 (0.5)	5.5 (0.5)	5.5 (0.5)	5.5 (0.5)
Fasting insulin, median (25, 75%), pmol/l^a^	71.8 (43.1–107.6)	64.6 (43.1–100.5)	64.6 (43.1–93.3)	64.6 (43.1–93.3)	64.6 (43.1–93.3)	64.6 (43.1–93.3)	71.8 (43.1–107.6)
HOMA-IR, median (25, 75%)^a^	2.8 (1.8–4.3)	2.6 (1.7–4.0)	2.5 (1.6–4.0)	2.5 (1.6–3.9)	2.5 (1.7–3.8)	2.6 (1.7–4.0)	2.8 (1.7–4.6)
Parental history of diabetes, no. (%)	104 (22.9)	363 (23.7)	633 (21.9)	809 (22.3)	580 (23.1)	190 (20.6)	76 (25.1)
White blood cell count, median (25, 75%), 10^3a^	6.3 (5.1–7.5)	5.9 (4.9–7.2)	5.8 (4.8–7.0)	5.6 (4.7–6.9)	5.6 (4.7–6.9)	5.7 (4.7–6.8)	5.6 (4.7–6.9)
Fibrinogen, median (25, 75%), mg/dl^a^	314 (277–362)	295 (261–342)	295 (260–336)	288 (255–327)	285 (255–324)	286 (254–323)	294 (261–333)
Physical activity index, mean (SD)^a^	6.7 (1.5)	6.9 (1.5)	7.0 (1.4)	7.1 (1.4)	7.2 (1.4)	7.2 (1.4)	7.2 (1.5)

*ABI* ankle-brachial index, *BMI* body mass index, *SBP* systolic blood pressure, *DBP* diastolic blood pressure, *TIA* transient ischemic attack, *CHD* coronary heart disease, *HDL* high density cholesterol, *HOMA-IR* homeostatic model assessment of insulin resistance

^a^Indicates statistically significant difference among ABI groups

**Fig. 1:**
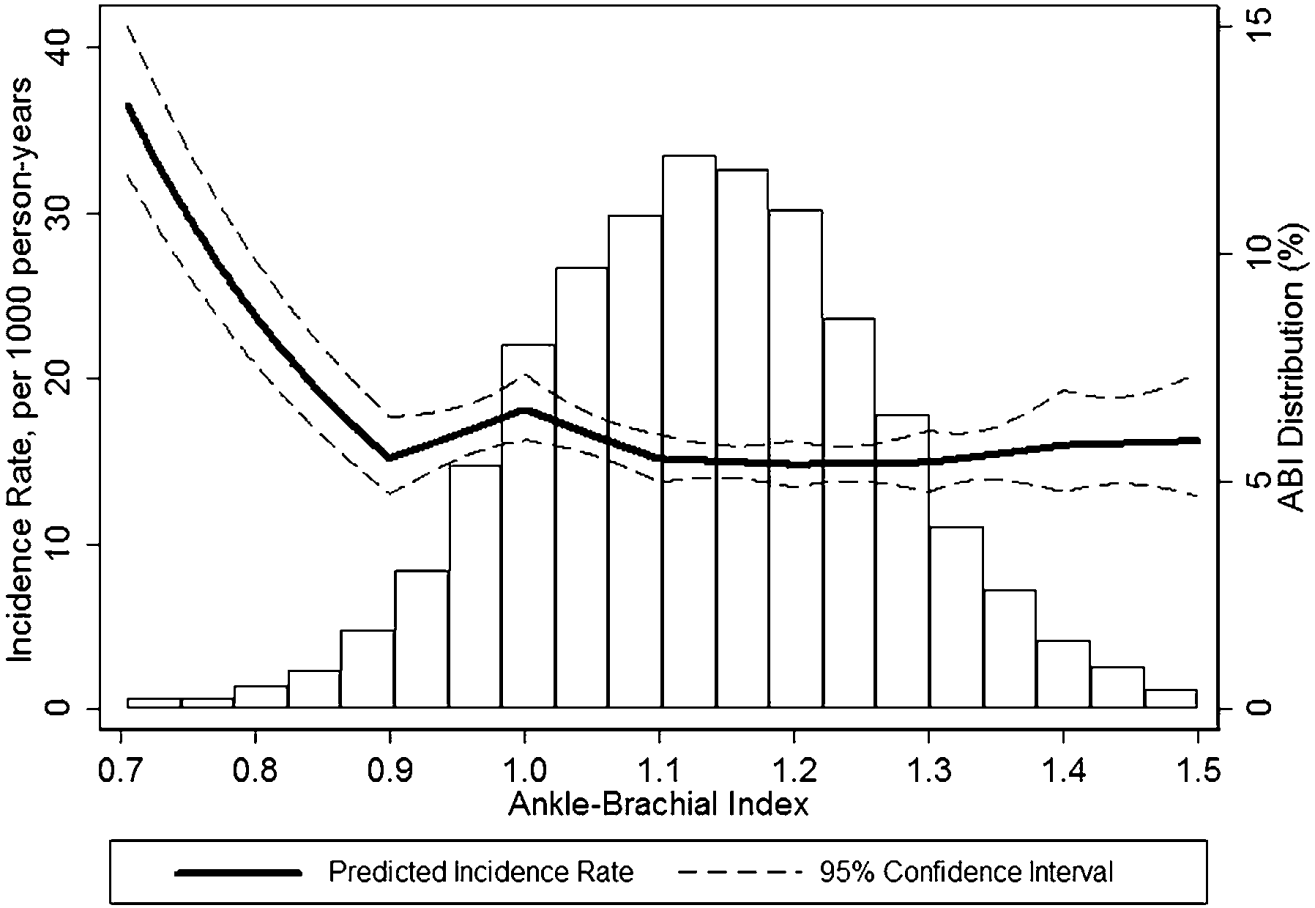
Demographically adjusted incidence rates of diabetes according to ABI and distribution of ABI. Graphed for 0.5–99.5 percentile of ABI values. Adjusted to mean age, white and male

A total of 3305 cases of incident diabetes were identified during a median of 21 years of follow-up (incidence rate 16.8 [95% CI 15.8–16.9] per 1000 person-years). Figure 1 shows demographically adjusted incidence rates of diabetes according to ABI at baseline. The incidence rates of diabetes were lowest in ABI 1.10–1.30 and increased below this range. The incidence rate of diabetes was similar or slightly higher in ABI > 1.30 compared to ABI 1.10–1.30.

In a demographically adjusted Cox regression model with ABI 1.10–1.20 as the reference, lower ABI categories were significantly associated with incident diabetes (hazard ratio [HR] 1.41 [95% CI 1.17–1.68] for ABI ≤ 0.90, 1.29 [1.15–1.45] for ABI 0.91–1.00, and 1.10 [1.00–1.22] for ABI 1.01–1.10, Model 1 in Table 2). When we further adjusted for other potential confounders including traditional cardiovascular risk factors, white blood cell count, and physical activity (Model 2 in Table 2), the associations for all lower ABI categories remained significant although it was borderline significant for ABI ≤ 0.90. The replacement of white blood cell count with fibrinogen did not make material difference (data not shown). After accounting for baseline fasting glucose (Model 3), the association remained marginally significant only in participants with ABI 0.91–1.00 (p value = 0.051). However, when we combined low ABI ≤ 0.90 and borderline low 0.91–1.00 groups, the association was statistically significant even in Model 3 (HR = 1.12 [95% CI 1.01–1.24], p value = 0.034). The associations between ABI ≤ 0.90 and ABI 0.91–1.00 with incident diabetes were slightly stronger than those in model 3 when we further adjusted for HOMA-IR in addition to model 2 (Model 4). For participants with ABI > 1.40, we observed slight but non-significant increase in the risk of incident diabetes compared to those with ABI 1.11–1.20 in Models 1 and 3. The association was largely consistent when we analyzed interview-based cases and visit-based cases separately (Additional file 1: Tables S1, S2). The model with physical activity as a time-varying covariate using visit 3 data showed similar results (data not shown).

**Table 2:** Hazard ratios of diabetes in different ABI categories

	ABI	≤0.90	0.91–1.00	1.01–1.10	1.11–1.20	1.21–1.30	1.31–1.40	>1.40
N	12,247	455	1529	2894	3633	2509	924	303
Number of events	3305	137	457	781	927	668	249	86
Model 1	HR	1.41	1.29	1.10	Ref	1.06	1.07	1.16
	95% CI	1.17–1.68	1.15–1.45	1.00–1.22	–	0.96–1.17	0.93–1.23	0.93–1.45
Model 2	HR	1.20	1.17	1.10	Ref	1.08	1.10	1.01
	95% CI	0.99–1.43	1.04–1.31	1.00–1.21	–	0.98–1.20	0.96–1.27	0.81–1.27
Model 3	HR	1.12	1.12	1.08	Ref	1.06	1.09	1.12
	95% CI	0.94-1.34	0.99–1.26	0.98–1.18	–	0.96–1.17	0.94–1.25	0.90–1.40
Model 4	HR	1.18	1.14	1.09	Ref	1.09	1.10	1.00
	95% CI	0.98–1.41	1.02–1.28	0.99–1.20	–	0.99–1.21	0.95–1.26	0.80–1.25

Model 1, adjusted for age, gender and race; Model 2, adjusted for age, gender, race, current and former drinking, current and former smoking, BMI, SBP, hypertension medication, HDL, total cholesterol, log (triglyceride), prevalent CHD, stroke or TIA, statin use, parental history of diabetes, log (white blood cell count) and Baecke physical activity index; Model 3, adjusted for baseline fasting glucose in addition to model 2; Model 4, adjusted for baseline log (HOMA-IR) in addition to model 2

To obtain reliable estimates in subgroup analyses, we dichotomized ABI at below and above 1.00. Given slight increase in the risk of diabetes in some models, those with ABI > 1.40 were excluded from this analysis. We observed significant difference in participants with vs. without history of stroke/TIA (HR 1.56 [95% CI 1.09–2.24] vs. 1.05 [0.95–1.15], p value for interaction = 0.034) and in those with vs. without hypertension (HR 0.96 [0.84–1.10] vs. 1.18 [95% CI 1.05–1.33], p value for interaction = 0.024) (Table 3). We also observed borderline significant difference between those with normal vs. impaired fasting glucose (HR 1.20 [95% CI 1.04–1.39] vs. 1.01 [0.90–1.14], p value for interaction = 0.067). The higher risk of diabetes was confirmed for both ABI categories of ≤0.90 and 0.91–1.00 in participants with normal fasting glucose, those without hypertension, and those with a history of stroke/TIA (Additional file 1: Tables S3, S4, S5).

**Table 3:** Hazard ratios of diabetes in different subgroups

Subgroup	HR and 95% CI ABI ≤ 1.00 vs 1.01–1.40	p value for interaction
All	1.07 (0.98, 1.17)	–
Gender		0.46
Male (5260)	1.13 (0.96–1.33)	
Female (6684)	1.05 (0.94–1.17)	
Race		0.89
White (9180)	1.08 (0.97–1.20)	
Black (2764)	1.06 (0.90–1.25)	
Age		0.35
≤55 (7193)	1.04 (0.92–1.17)	
>55 (4751)	1.13 (0.98–1.30)	
Current smoking		0.77
No (8887)	1.07 (0.96–1.19)	
Yes (3057)	1.10 (0.93–1.30)	
Prevalent CHD		0.41
No (11,465)	1.08 (0.99–1.19)	
Yes (479)	0.89 (0.58–1.39)	
History of stroke/TIA		0.034
No (11,444)	1.05 (0.95–1.15)	
Yes (500)	1.56 (1.09–2.24)	
Hypertension		0.024
No (8923)	1.18 (1.05–1.33)	
Yes (3622)	0.96 (0.84–1.10)	
Family history of diabetes		0.37
No (9625)	1.04 (0.93–1.16)	
Yes (2679)	1.14 (0.97–1.34)	
Baseline FBG 5.6–6.9 mmol/l		0.067
No (6895)	1.20 (1.04–1.39)	
Yes (5049)	1.01 (0.90-1.14)	
Leg pain while walking		0.58
No (9606)	1.05 (0.95–1.17)	
Yes (2338)	1.12 (0.94–1.33)	

N = 11,944

Excluded ABI > 1.4 and adjusted for age, gender, race, current and former drinking, current and former smoking, BMI, SBP, hypertension medication, HDL, total cholesterol, log (triglyceride), prevalent CHD, stroke or TIA, statin use, parental history of diabetes, log (white blood cell count), Baecke physical activity index and fasting glucose

## Discussion

To our knowledge, this is the first study to examine the association between ABI and the future risk of diabetes. We found that low and borderline low ABI (≤1.0) was associated with a moderately increased risk of diabetes. The association was independent of other atherosclerotic cardiovascular diseases, physical activity, and other potential confounders. Further adjustment for fasting glucose levels attenuated the association, but ABI ≤ 1.0 showed significantly higher risk of diabetes compared to ABI 1.11–1.20 even in this model. Largely similar results were observed in demographic and clinical subgroups, although the associations tended to be stronger in participants without hypertension, those with normal fasting glucose, and those with history of stroke/TIA compared to their counterparts.

In terms of potential mechanisms, as we hypothesized, impaired physical activity related to PAD may play a role. However, in our study, the ABI-diabetes relationship remained significant after accounting for physical activity. Yet, we need to bear in mind that physical activity was based on self-report [25]. Also, there are a few other potential mechanisms linking low ABI to future diabetes risk. ABI is a marker of systematic atherosclerosis [26], and participants with low ABI indeed had worse cardiovascular risk profiles in our study. Several traditional cardiovascular risk factors such as hypertension, smoking, and dyslipidemia are known to be related to high risk of developing diabetes [27–29]. Thus, we rigorously adjusted for these traditional risk factors but still observed significant associations between ABI and risk of diabetes. Also, endothelial dysfunction, an early condition of atherosclerosis, may be a contributor. It has been shown that delayed insulin delivery can occur due to endothelial dysfunction [30]. Indeed, endothelial dysfunction is associated with future risk of type 2 diabetes in a few studies [31, 32]. In addition, shared pathophysiology such as inflammation is known for atherosclerosis and insulin resistance [33, 34]. Although chronic inflammation can be a common ground for development of both atherosclerosis and diabetes [33–36], our results were not altered with adjustment for white blood cell count or fibrinogen.

We found that the association between low and borderline low ABI (≤1.00) and risk of diabetes tended to be stronger in participants without hypertension, those with normal fasting glucose, and those with a history of stroke/TIA as compared to their counterparts. We are not necessarily sure about mechanisms behind these suggestive interactions, but there may be a few potential explanations for null association in participants with hypertension or elevated glucose level. Many of those with hypertension were treated with antihypertensive medications (72%), which might confound the ABI-diabetes association to null among hypertensive patients. Indeed, renin-angiotensin system inhibitors are reported to reduce the risk of diabetes [37], whereas diuretics and beta blockers may contribute to increased risk of developing diabetes [38]. For people with impaired fasting glucose, who are known to have reduced insulin sensitivity and β cell dysfunction [39], a mild single predictor such as ABI may not considerably contribute to discriminating their diabetes risk since they are already at high risk of diabetes. Nonetheless, we need to keep in mind that we have tested multiple subgroups without a priori hypothesis, and thus, our subgroup analysis should be interpreted as hypothesis-generating.

### Clinical and research implications of the study

Although our findings need to be confirmed in other settings, there may be a few clinical and research implications from our study. Our study demonstrates future diabetes as another adverse clinical consequence of PAD in addition to its known complications such as other cardiovascular diseases and impaired functional status [3, 4, 7, 26, 40–44]. This finding is important given the adverse outcomes associated with diabetes [44–49]. Since some clinical guidelines recommend screening of PAD using the ABI in individuals with advanced age and/or traditional cardiovascular risk factors [1, 50], attention should be given to monitoring glucose levels among individuals with low or borderline low ABI even though their fasting glucose is within the normal range at baseline. If our results are replicated, it would be worth assessing whether PAD-specific interventions (e.g., supervised exercise [51]) have beneficial effects on glucose metabolism and whether other measures of subclinical atherosclerosis (e.g., carotid intra-media thickness or coronary artery calcium) are related to incident diabetes.

## Limitations of the study

Our study has several limitations. First, ABI was measured once for a randomly selected leg at baseline. The prevalence of low ABI may be underestimated as a result of missing low ABI in the opposite leg in some participants. Second, there were 15 years of follow-up where incident diabetes cases were solely based on self-report (interview-based definition). However, as aforementioned, the association was largely consistent for visit-based and interview-based diabetes. Third, we were not able to adjust for baseline hemoglobin A1c as it was not available at visit 1. Fourth, our study participants were 45–64 years old at baseline, and thus the generalization of our results to adults in other age ranges should be done with caution. Finally, like other observational studies, residual confounding cannot be denied.

## Conclusions

Low ABI (≤1.00) was modestly but independently associated with increased risk of future diabetes in community-based middle-aged populations. Although future studies are needed to confirm our findings and investigate potential mechanisms, our study suggests that clinical attention should be given to glucose trajectory in people with low or borderline low ABI.

## Additional file

**Additional file 1.** Online supplementary tables.

## Abbreviations

PAD: peripheral artery disease
ABI: ankle-brachial index
HR: hazard ratio
CI: confidence interval
ARIC: the Atherosclerosis Risk in Communities Study
CHD: coronary heart disease
TIA: transient ischemic attack
ANOVA: analysis of variance
BMI: body mass index
SBP: systolic blood pressure
DBP: diastolic blood pressure
HDL: high density cholesterol
FBG: fasting blood glucose
HOMA-IR: homeostatic model assessment of insulin resistance
