# Supplementary material for: Ankle-brachial index and incident diabetes mellitus: the atherosclerosis risk in communities (ARIC) study
Source: Cardiovasc Diabetol. 2016 Dec 7;15:163. doi: 10.1186/s12933-016-0476-4 (PMC5142100; doi:10.1186/s12933-016-0476-4)
Supplement: Supplementary file 1 — Additional file 1. Online supplementary tables. [file 12933_2016_476_MOESM1_ESM.docx]

| **Table S1 Hazard Ratios of Interview-based Definition of Diabetes in Different ABI Categories** | | | | | | | | |
| --- | --- | --- | --- | --- | --- | --- | --- | --- |
|  | **ABI** | **≤0.90** | **0.91-1.00** | **1.01-1.10** | **1.11-1.20** | **1.21-1.30** | **1.31-1.40** | **>1.40** |
| **N** | **11,292** | 390 | 1,397 | 2,668 | 3,373 | 2,320 | 861 | 283 |
| **Number of events** | **2,959** | 118 | 414 | 698 | 841 | 596 | 217 | 75 |
| **Model 1** | **HR** | 1.39 | 1.29 | 1. 08 | Ref | 1.05 | 1.02 | 1.12 |
|  | **95%CI** | 1.15-1.69 | 1.14-1.45 | 0.98-1.20 | -- | 0.94-1.16 | 0.88-1.18 | 0.88-1.42 |
| **Model 2** | **HR** | 1.21 | 1.16 | 1.08 | Ref | 1.06 | 1.04 | 0.98 |
|  | **95%CI** | 1.00-1.47 | 1.03-1.30 | 0.97-1.19 | -- | 0.95-1.18 | 0.89-1.20 | 0.77-1.24 |
| **Model 3** | **HR** | 1.16 | 1.12 | 1.05 | Ref | 1.04 | 1.03 | 1.07 |
|  | **95%CI** | 0.96-1.41 | 0.99-1.26 | 0.95-1.16 | -- | 0.94-1.16 | 0.88-1.19 | 0.84-1.35 |
| **Model 4** | **HR** | 1.17 | 1.14 | 1.07 | Ref | 1.07 | 1.04 | 0.96 |
|  | **95%CI** | 0.96-1.42 | 1.01-1.29 | 0.96-1.18 | -- | 0.96-1.19 | 0.89-1.20 | 0.76-1.22 |
| Model 1: adjusted for age, gender and race; Model 2: adjusted for age, gender, race, current and former drinking, current and former smoking, BMI, SBP, hypertension medication, HDL, total cholesterol, log(triglyceride), prevalent CHD, stroke or TIA, statin use, parental history of diabetes, log(white blood cell count) and Baecke physical activity index; Model 3: adjusted for baseline fasting glucose in addition to model 2; Model 4: adjusted for baseline log(HOMA-IR) in addition to model 2. | | | | | | | | |

| **Table S2 Hazard Ratios of Visit-based Definition of Diabetes in Different ABI Categories** | | | | | | | | |
| --- | --- | --- | --- | --- | --- | --- | --- | --- |
|  | **ABI** | **≤0.90** | **0.91-1.00** | **1.01-1.10** | **1.11-1.20** | **1.21-1.30** | **1.31-1.40** | **>1.40** |
| **N** | **11,858** | 432 | 1,481 | 2,798 | 3,525 | 2,441 | 889 | 292 |
| **Number of events** | **1,385** | 59 | 185 | 332 | 378 | 287 | 110 | 34 |
| **Model 1** | **HR** | 1.51 | 1.26 | 1. 13 | Ref | 1.12 | 1.12 | 1.02 |
|  | **95%CI** | 1.15-1.99 | 1.05-1.50 | 0.98-1.31 | -- | 0.96-1.31 | 0.90-1.38 | 0.72-1.45 |
| **Model 2** | **HR** | 1.23 | 1.15 | 1.14 | Ref | 1.18 | 1.16 | 1.01 |
|  | **95%CI** | 0.94-1.63 | 0.96-1.38 | 0.98-1.32 | -- | 1.01-1.37 | 0.93-1.43 | 0.71-1.44 |
| **Model 3** | **HR** | 1.26 | 1.05 | 1.02 | Ref | 1.06 | 1.08 | 0.99 |
|  | **95%CI** | 0.96-1.67 | 0.88-1.26 | 0.88-1.19 | -- | 0.91-1.24 | 0.87-1.33 | 0.70-1.42 |
| **Model 4** | **HR** | 1.24 | 1.08 | 1.10 | Ref | 1.15 | 1.13 | 0.97 |
|  | **95%CI** | 0.94-1.64 | 0.90-1.29 | 0.95-1.28 | -- | 0.98-1.34 | 0.91-1.40 | 0.68-1.38 |
| Model 1: adjusted for age, gender and race; Model 2: adjusted for age, gender, race, current and former drinking, current and former smoking, BMI, SBP, hypertension medication, HDL, total cholesterol, log(triglyceride), prevalent CHD, stroke or TIA, statin use, parental history of diabetes, log(white blood cell count) and Baecke physical activity index; Model 3: adjusted for baseline fasting glucose in addition to model 2; Model 4: adjusted for baseline log(HOMA-IR) in addition to model 2. | | | | | | | | |

| **Table S3 Hazard Ratios of Diabetes in Baseline Fasting Glucose<5.6 mmol/L and 5.6-6.9 mmol/L population** | | | | | | | | |
| --- | --- | --- | --- | --- | --- | --- | --- | --- |
|  | **ABI** | **≤0.90** | **0.91-1.00** | **1.01-1.10** | **1.11-1.20** | **1.21-1.30** | **1.31-1.40** | **>1.40** |
| **N** | **7,080** | 257 | 880 | 1,707 | 2,093 | 1,419 | 539 | 185 |
| **Number of events** | **1,242** | 53 | 178 | 297 | 349 | 241 | 88 | 36 |
|  |  |  |  |  |  |  |  |  |
| **<5.6 mmol/L** | **HR** | 1.33 | 1.27 | 1.06 | Ref | 1.06 | 1.03 | 1.03 |
|  | **95%CI** | 0.99-1.79 | 1.06-1.53 | 0.90-1.23 | -- | 0.90-1.25 | 0.82-1.31 | 0.73-1.45 |
| **N** | **5,167** | 198 | 649 | 1,187 | 1,540 | 1,090 | 385 | 118 |
| **Number of events** | **2,063** | 84 | 279 | 484 | 578 | 427 | 161 | 50 |
| **5.6-6.9 mmol/L** | **HR** | 1.09 | 1.07 | 1.13 | Ref | 1.09 | 1.17 | 1.14 |
|  | **95%CI** | 0.87-1.38 | 0.93-1.24 | 1.00-1.28 | -- | 0.96-1.23 | 0.98-1.40 | 0.85-1.53 |
| Adjusted for age, gender, race, current and former drinking, current and former smoking, BMI, SBP, hypertension medication, HDL, total cholesterol, log(triglyceride), prevalent CHD, stroke or TIA, statin use, parental history of diabetes, log(white blood cell count) and Baecke physical activity index. | | | | | | | | |

| **Table S4 Hazard Ratios of Diabetes in Study Participants with and without Hypertension at Baseline** | | | | | | | | |
| --- | --- | --- | --- | --- | --- | --- | --- | --- |
|  | **ABI** | **≤0.90** | **0.91-1.00** | **1.01-1.10** | **1.11-1.20** | **1.21-1.30** | **1.31-1.40** | **>1.40** |
| **N** | **8,539** | 265 | 1,023 | 1,961 | 2,556 | 1,837 | 680 | 217 |
| **Number of events** | **1,954** | 77 | 259 | 423 | 568 | 425 | 154 | 48 |
|  |  |  |  |  |  |  |  |  |
| **No hypertension** | **HR** | 1.27 | 1.15 | 0.99 | Ref | 1.01 | 1.11 | 0.98 |
|  | **95%CI** | 0.99-1.61 | 0.99-1.34 | 0.88-1.13 | -- | 0.89-1.15 | 0.93-1.33 | 0.73-1.32 |
| **N** | **3,708** | 190 | 506 | 933 | 1,077 | 672 | 244 | 86 |
| **Number of events** | **1,351** | 60 | 198 | 358 | 359 | 243 | 95 | 38 |
| **Hypertension** | **HR** | 1.01 | 1.13 | 1.22 | Ref | 1.13 | 1.10 | 1.38 |
|  | **95%CI** | 0.77-1.33 | 0.94-1.34 | 1.05-1.41 | -- | 0.96-1.33 | 0.88-1.39 | 0.98-1.93 |
| Adjusted for age, gender, race, current and former drinking, current and former smoking, BMI, HDL, total cholesterol, log(triglyceride), prevalent CHD, stroke or TIA, statin use, parental history of diabetes, log(white blood cell count), Baecke physical activity index and fasting glucose. | | | | | | | | |

| **Table S5 Hazard Ratios of Diabetes in Study Participants with and without History of Stroke or TIA at Baseline** | | | | | | | | |
| --- | --- | --- | --- | --- | --- | --- | --- | --- |
|  | **ABI** | **≤0.90** | **0.91-1.00** | **1.01-1.10** | **1.11-1.20** | **1.21-1.30** | **1.31-1.40** | **>1.40** |
| **N** | **11,732** | 422 | 1,461 | 2,771 | 3,482 | 2,410 | 898 | 288 |
| **Number of events** | **3,141** | 123 | 431 | 744 | 882 | 635 | 243 | 83 |
|  |  |  |  |  |  |  |  |  |
| **No history of stroke/TIA** | **HR** | 1.06 | 1.10 | 1.08 | Ref | 1.05 | 1.10 | 1.13 |
|  | **95%CI** | 0.87-1.28 | 0.98-1.24 | 0.98-1.19 | -- | 0.95-1.16 | 0.96-1.27 | 0.90-1.41 |
| **N** | **515** | 33 | 68 | 123 | 151 | 99 | 26 | 15 |
| **Number of events** | **164** | 14 | 26 | 37 | 45 | 33 | 6 | 3 |
| **History of stroke/TIA** | **HR** | 1.86 | 1.30 | 0.94 | Ref | 1.12 | 0.71 | 0.92 |
|  | **95%CI** | 0.98-3.50 | 0.78-2.17 | 0.60-1.47 | -- | 0.70-1.78 | 0.29-1.73 | 0.28-3.02 |
| Adjusted for age, gender, race, current and former drinking, current and former smoking, BMI, SBP, hypertension medication, HDL, total cholesterol, log(triglyceride), prevalent CHD, statin use, parental history of diabetes, log(white blood cell count), Baecke physical activity index and fasting glucose. | | | | | | | | |
